# Predictive divergence in machine learning models for clinical mortality risk: A multicohort study of covid-19 patients

**DOI:** 10.1371/journal.pone.0344354

**Published:** 2026-03-06

**Authors:** Júlia Chaves Neuenschwander Magalhães, Alexandre Dias Porto Chiavegatto Filho

**Affiliations:** Department of Epidemiology, School of Public Health, University of São Paulo, São Paulo, São Paulo, Brazil; Universidade Federal de Minas Gerais, BRAZIL

## Abstract

**Background:**

Machine learning (ML) algorithms are increasingly used in healthcare to support clinical decision-making. While models with similar overall performance are often considered interchangeable for deployment, they may produce divergent predictions, a phenomenon known as algorithmic multiplicity. In such cases, the choice of algorithm may introduce bias. This study investigates the impacts of algorithmic multiplicity in mortality prediction and assesses the influence of patient characteristics on model decisions.

**Methods:**

A cohort of 4,337 adult patients (≥18 years) with RT-PCR–confirmed covid-19 from five tertiary care hospitals in Brazil was followed from March to August 2020. Five popular ML models for structured data were trained on demographic and laboratory data collected at early hospital admission to predict in-hospital mortality. Model performance, feature importance, and algorithmic prediction similarity were evaluated. Feature distributions were compared between patients correctly or incorrectly classified by all models using paired t-tests or Mann–Whitney U tests, as applicable, at the 5% significance level. Subgroup performance differences were assessed using 10-fold cross-validation applied to five k-means–delineated clusters, compared by one-way ANOVA. Within-cluster predictive divergence was assessed within a 95% confidence interval.

**Results:**

All models achieved high overall predictive performance (µ = 0.855, σ² = 0.0072). However, the comparison of individual-level predictions revealed substantial heterogeneity, with pairwise prediction correlations ranging from R² = 0.56 to 0.80. Unsupervised k-means clustering identified five clinically distinct patient subgroups with mortality rates ranging from 22% to 80%, within which model performance varied significantly (F = 73.18, p < 0.001). Notably, TabPFN and LightGBM showed superior performance in the “Anemia” cluster, whereas TabPFN underperformed in the “Immunodeficient” cluster (95% CI).

**Conclusions:**

This study demonstrates that ML models with similar overall performance can yield substantially divergent predictions at both the individual and subgroup levels, and that no single algorithm consistently outperforms others across all patient subgroups. These findings highlight the limitations of relying solely on global performance metrics and underscore the need for context-aware evaluation of ML models in heterogeneous clinical populations.

## Introduction

In recent years, rapid advances in artificial intelligence (AI), especially in its machine learning (ML) subfield, have driven the increasing adoption of these technologies in medicine [1], particularly in the development of computer vision systems for medical imaging analysis [2]. In parallel, there has been growing interest in models based on structured variables, which leverage routinely collected demographic and laboratory data to provide cost-effective and highly scalable solutions in hospital settings [3]. In the context of mortality prediction, such models may support clinical decision-making by enabling early risk stratification, identifying patients who may require closer monitoring or earlier escalation of care, and informing the prioritization of clinical resources in high-risk settings. The scale of this adoption is evident in the Artificial Intelligence Index Report 2025, which documents a fivefold increase in published clinical trials evaluating AI-based systems for clinical decision support between 2019 and 2024 [4].

These models are no longer confined to research settings and are increasingly being deployed in real-world clinical practice. In a 2024 survey of 67 health systems across the United States, 90% of respondents reported deploying AI tools for imaging and radiology in at least limited clinical settings, while 67% had implemented models for early sepsis detection and 52% had deployed models to predict hospital readmission risk [5]. However, this widespread use of ML in healthcare has also raised significant fairness and ethical concerns, particularly given the high-stakes nature of clinical decision-making [5,6].

As algorithmic performance improves, selecting an appropriate model becomes more challenging. Accuracy alone is often insufficient, as multiple models may achieve statistically indistinguishable performance. This situation reflects an established yet still underexplored phenomenon: algorithmic multiplicity. Originally introduced by Breiman (2001), algorithmic multiplicity describes scenarios in which multiple models with comparable predictive performance yield different predictions for the same instances [7,8]. Formally, let $h_{\text{0}}\;$ denote a base classifier and ∈ a tolerance level. The set of competing classifiers is defined as,


Sϵ(h0):={h:R(h)≤R(h0)+ϵ}

where $R(h_{\text{0}})$ denotes the error rate of the base classifier. A prediction is said to exhibit algorithmic multiplicity if there exists a model $h_{\text{1}}\in S_{\epsilon }(h_{\text{0}})$ such that $h_{\text{1}}(x_{i})\neq h_{\text{0}}(x_{i})$ for at least one training instance $x_{i}$ [8].

This definition indicates that algorithmic multiplicity is not merely a theoretical artifact but has practical consequences. The indeterminacy resulting from multiplicity can lead to arbitrary and unfair decisions, since given equivalent performance, the very choice of an algorithm may introduce bias [6,8,9]. This is particularly concerning in large-scale applications, as such arbitrariness might result in severe outcomes for individuals or groups systematically disadvantaged by the chosen model [10]. On the other hand, understanding this multiplicity paves the way for selecting models based on criteria such as interpretability and fairness, without significantly compromising accuracy, thus allowing for greater flexibility in model choice [6].

In this context, the present study investigates the effects of algorithmic multiplicity using covid-19 mortality prediction as a clinically relevant and data-rich case study. Data from Brazilian hospitals provides a particularly suitable context due to the country’s large geographic extent and demographic diversity, yielding a heterogeneous patient population. Five state-of-the-art ML algorithms reported in the literature were evaluated [2,11], each trained with samples of varying sizes. The objectives were to compare predictive performance, identify consistent error patterns, and assess the influence of sample characteristics on predictive divergence.

Although the empirical analysis focuses on covid-19 mortality, the methodological insights regarding predictive variability, subgroup-specific errors, and model selection under algorithmic multiplicity apply to a broad range of clinical prediction tasks. By characterizing algorithmic multiplicity in a large-scale clinical dataset, this work aims to contribute to a deeper understanding of predictive divergence in ML systems and may inform the development of more robust, equitable, and transparent clinical decision support tools.

## Methods

### Data source and study design

A multicohort retrospective study, aiming to investigate the effects of algorithmic multiplicity on mortality prediction for covid-19 in hospitals across Brazil, was conducted with 4,377 adult patients (≥18 years) with RT-PCR–confirmed covid-19, who were followed between March and August 2020. Data were obtained from the IACOV-BR database, which integrates records from five hospitals, with cohort sizes ranging from 247 to 1,776 patients. Only anonymized data, originally collected for clinical care and limited to patients who had already been discharged, were included. These data were accessed for research purposes on June 6, 2024. The study received ethical approval from the University of São Paulo’s Institutional Review Board (IRB), in accordance with the resolutions of the National Health Council (CNS) and National Research Ethics Committee (CONEP), under reference number 32872920.4.1001.5421, which included a waiver of consent. This approval also covered the utilization of data and collaboration with all hospitals participating in the IACOV-BR network.

### Data preprocessing

The dataset was split using random sampling stratified by the outcome, with 70% allocated for training and 30% for testing. Categorical variables with two or more categories were one-hot encoded into sets of dummy variables. Continuous variables were normalized by z-score transformation, using the mean and standard deviation calculated from the training set. Columns with more than 90% correlation were eliminated, as were variables with more than 90% missing data. The remaining missing values were handled by median imputation.

Given the imbalanced nature of the dataset, class balancing was performed using the Random Oversampling technique, applied to the training set. SMOTE and Borderline SMOTE were also tested but were discarded as they did not improve predictive performance. Hyperparameters were selected through Bayesian optimization using HyperOpt, coupled with 10-fold cross-validation.

### Model development and evaluation

Five popular models for structured data, XGBoost, LightGBM, Catboost, Random Forest, and TabPFN, were trained on demographic and laboratory data collected during early hospital admission (within ±24 h from RT-PCR exam) to predict in-hospital mortality. A total of 22 predictors were included: age, sex, heart rate, respiratory rate, systolic pressure, diastolic pressure, mean arterial pressure, temperature, hemoglobin, platelets, hematocrit, erythrocytes, mean corpuscular hemoglobin (MCH), red cell distribution width (RDW), mean corpuscular volume (MCV), leukocytes, neutrophils, lymphocytes, basophils, eosinophils, monocytes, and C-reactive protein (CRP). These features were selected based on their routine availability during hospital admissions, including in low-resource settings, and their demonstrated predictive performance [12].

The predictive performance was measured through the area under the receiver operating characteristic curve (AUC). Additionally, accuracy, precision, recall, and F1-score were calculated. Feature importance was assessed using the Shapley Additive Explanations (SHAP) method for XGBoost, Catboost, LightGBM, and Random Forest. TabPFN interpretability was constrained due to tool compatibility, necessitating the use of proxy interpretability via Shapley Interaction Quantification (SHAP-IQ).

### Cross-model prediction similarity

The similarity among the algorithms was first evaluated through paired distribution plots of the probabilistic predictions, on which the R² statistic was calculated. Additionally, histograms of the probability distributions for each model were analyzed to gain insights into the classification behavior of each algorithm.

### Misclassification patterns

To investigate misclassification patterns associated with sample characteristics, two patient groups were defined: patients unanimously correctly or incorrectly classified by all five models. The distribution of predictor variable values was compared between these groups. Variables with homoscedasticity confirmed by Levene’s test were analyzed using a paired t-test, while those with unequal variance between groups were compared using the non-parametric Mann–Whitney U test. Variables that rejected the null hypothesis at a 5% significance level were considered statistically significant. The findings were then compared with the set of variables deemed important for model decisions, as calculated using Shapley values.

### Subgroup predictive performance

To assess algorithm performance across patient subgroups, the training set was partitioned into five clusters using the unsupervised k-means algorithm. The number of clusters was selected based on the elbow method, through minimization of the weighted cumulative sum of squares (WCSS). These clusters were characterized by using the ten most important predictors on average across all algorithms, as determined by SHAP, and visualized through a radar chart. The clustered data were used to re-train all five models using 10-fold cross-validation. The resulting AUC was compared across subgroups by a one-way ANOVA test. Within-group predictive divergence was analyzed in a 95% confidence interval.

All analyses were conducted using Python 3, with ML models implemented through Scikit-learn.

## Results

### Demographic characteristics of the sample

As presented in Table 1, the analyzed sample consisted of 4,377 adult patients with RT-PCR confirmed covid-19 admitted across five different hospitals. The list of hospitals and their respective locations is presented in S1 Table. The mean age of participants was 56.1 years (SD = 17.2), and 55.1% were male. 15.5% of participants reported ethnic identity, with 11.5% identifying as White and 3.9% as Black, Mixed, or Asian. Patients who died during the study period were, on average, older (65 years vs 52 years for survivors) and more likely to be male (60.7% vs 52% among survivors).

**Table 1:** Demographic characteristics of the sample by hospital and outcome.

Hospital	Death – Yes	Death – No	Total
**Number of patients, n (%)**			
Hospital 1 (S)	47 (10.3)	409 (89.6)	456 (100)
Hospital 2 (NE)	94 (6.9)	1265 (93.0)	1359 (100)
Hospital 3 (SE)	738 (41.5)	1038 (58.5)	1776 (100)
Hospital 4 (N)	38 (15.3)	209 (84.6)	247 (100)
Hospital 5 (CW)	328 (60.8)	211 (39.1)	539 (100)
**Total**	**1245 (28.4)**	**3132 (71.6)**	**4377 (100)**
**Age, mean (STD)**			
Hospital 1 (S)	83.5 (8.3)	57.4 (16.7)	60.1 (17.9)
Hospital 2 (NE)	73.9 (14.7)	47.5 (17.0)	49.4 (18.2)
Hospital 3 (SE)	64.0 (14.2)	56.2 (14.9)	59.4 (15.1)
Hospital 4 (N)	72.5 (12.9)	53.7 (16.1)	56.6 (17.0)
Hospital 5 (CW)	61.3 (15.9)	52.8 (13.9)	57.9 (15.7)
**Total**	**65.0 (15.3)**	**52.4 (16.6)**	**56.1 (17.2)**
**Male, %**			
Hospital 1 (S)	53.2	58.4	57.8
Hospital 2 (NE)	52.1	43.1	43.7
Hospital 3 (SE)	62.2	59.7	60.7
Hospital 4 (N)	61.3	55.5	55.8
Hospital 5 (CW)	60.7	63.5	62.1
**Total**	**60.7**	**52.8**	**55.0**
**Color – White, %**			
Hospital 1 (S)	95.7	92.6	92.9
Hospital 5 (CW)	25.0	0.0	15.2
**Color – Black/Mixed/Asian, %**			
Hospital 1 (S)	2.1	1.2	1.3
Hospital 5 (CW)	50.3	0.4	30.7

### Model performance and prediction agreement

To assess baseline predictive performance and the impact of site-specific heterogeneity, we compared algorithmic performance across hospitals using both locally trained and aggregated models.

Table 2 shows the AUC results by algorithm with local training in each hospital, as well as for training with aggregated data from all hospitals. The list of hyperparameters used for the aggregated data training, selected via Bayesian Optimization using HyperOpt coupled with 10-fold cross-validation, can be seen in S2 Table.

**Table 2:** Algorithmic performance (AUC) by hospital.

Hospital	n	Death (%)	Random Forest	XGBoost	LightGBM	Catboost	TabPFN
**Hospital 1**	456	47 (10.3)	0.89	0.88	0.89	0.89	0.90
**Hospital 2**	1359	94 (6.9)	0.87	0.89	0.86	0.90	0.82
**Hospital 3**	1776	738 (41.5)	0.69	0.69	0.67	0.69	0.72
**Hospital 4**	247	38 (15.3)	0.85	0.85	0.86	0.87	0.87
**Hospital 5**	539	328 (60.8)	0.73	0.67	0.67	0.68	0.73
**All**	**4377**	**1245 (28.4)**	**0.86**	**0.85**	**0.86**	**0.85**	**0.86**

Regarding the site-specific results, in absolute terms, TabPFN achieved the best performance, with the highest AUC values in four of the five cases (80%). Sample size did not show a clear association with prediction quality, whereas higher mortality rates were associated with lower AUC values on average.

As for aggregate performance metrics, they were largely comparable across algorithms both in terms of AUC (Table 2) and accuracy, precision, recall, and F1-score (S3 Table). However, as similar performance metrics do not necessarily imply similar prediction behavior, we proceeded to examine the concordance and distribution of probabilistic predictions across models.

**Fig 1:**
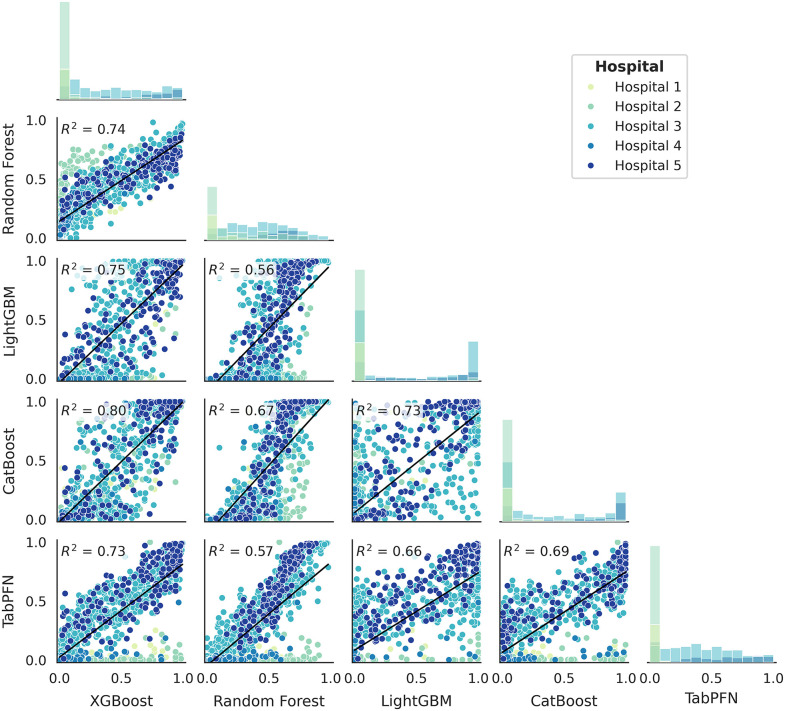
Probabilistic predictions correlation. Matrix of pairwise scatter plots showing the correlation between predicted probabilities of in-hospital mortality generated by the different machine-learning models (XGBoost, Random Forest, LightGBM, and Catboost). Each point represents an individual patient, colored by hospital. Solid lines indicate fitted linear regression models, with the corresponding coefficient of determination (R²) reported in each panel. Diagonal panels display histograms of predicted probability distributions for each model.

Fig 1 illustrates the similarity between models. The diagonal panels display histograms showing the individual distributions of predictions. It can be noted that Random Forest tends to generate more intermediate values compared to the other models, which show a greater propensity for extreme values. This characteristic is especially evident in LightGBM, which demonstrates a strong prevalence of predictions close to zero or one.

The lower panels contain scatter plots that illustrate the linear relationship between pairs of models, with a regression line in blue and an annotation of the R² statistic for each model pair. This analysis reveals that the highest correlation occurs between XGBoost and Catboost, while the lowest is observed between Random Forest and LightGBM. A notable discrepancy is observed in the frequency of probabilities close to zero for patients from Hospital 2, which can be attributed to its lower mortality rate (6.9%) (Table 2). Greater distribution dispersion is evident for pairs of boosting-based models (XGBoost, LightGBM, and Catboost), while comparisons involving XGBoost and Random Forest, as well as Random Forest and TabPFN, show a more concentrated distribution. This is surprising, as it does not align with architectural similarities. These results highlight important differences in the distributions and prediction patterns among the algorithms, revealing significant predictive divergence even when the variance in predictive performance is minimal.

### Error pattern analysis

Given the observed divergence in probabilistic predictions despite comparable performance metrics, we next investigated whether misclassifications were associated with specific patient characteristics.

**Fig 2:**
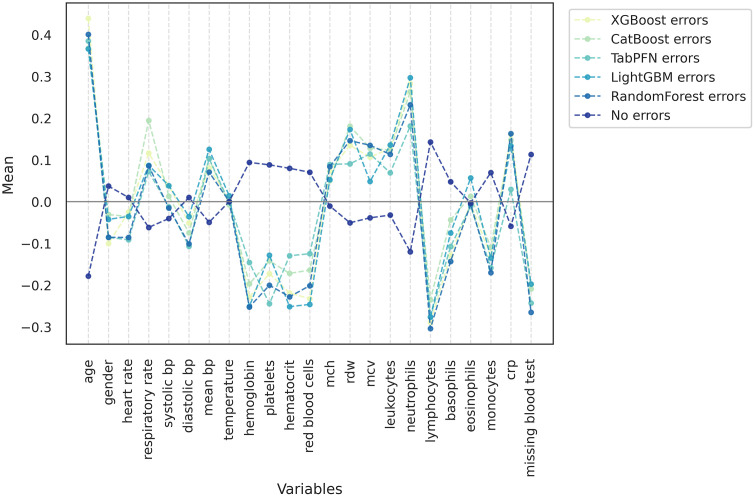
Profile graphs by error group. Profile plot showing the mean standardized value of each variable among patients misclassified by each machine-learning model. A reference group of correctly classified patients (“No errors”) is included for comparison. All variables were normalized using z-score transformation; values above and below zero indicate higher or lower mean levels relative to the overall cohort.

Fig 2 presents the patient profiles stratified by error group, based on the vector of group means derived from variables normalized via z-score transformation. The expected value for this vector corresponds to the null vector (represented by the grey line), a consequence of the transformation’s definition as z = (x – μ)/σ. Variables whose values lie close to this line are evenly distributed across groups and, thus, do not substantially contribute to the differentiation among error profiles. In contrast, variables that deviate from this pattern may be associated with increased susceptibility to errors in each algorithm.

It can be noted that, although there are variations in the error profiles across different models, they tend to follow a similar pattern, which contrasts with the profile observed in patients for whom no model produced errors. These observations suggest that certain population-level characteristics may be associated with a greater propensity for algorithm misclassification. Even so, certain variables exhibit different levels of influence across algorithms. For instance, respiratory rate appears to have a greater impact on the predictions generated by Catboost, whereas platelet count contributes less significantly to the predictions made by LightGBM, when compared to the other models.

**Fig 3:**
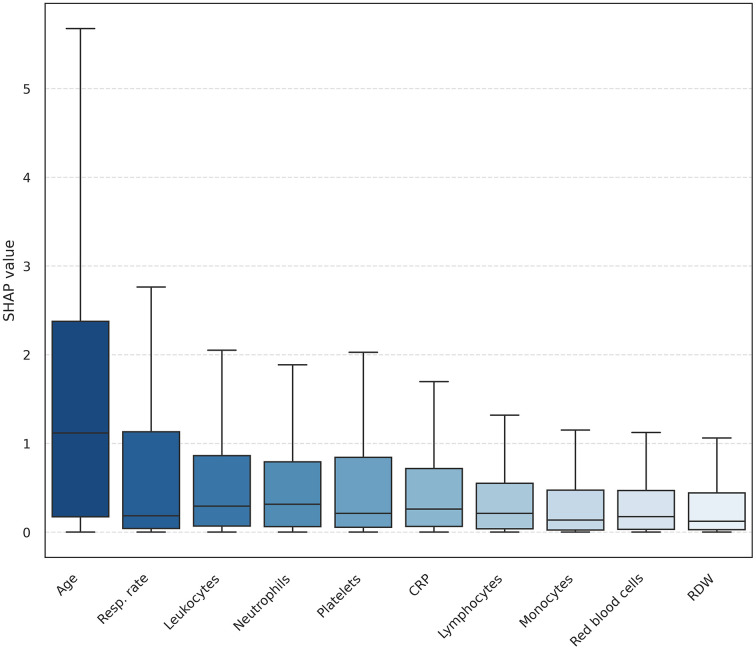
Variable importance boxplots. Boxplots showing feature importance estimated using Shapley Additive Explanations (SHAP) for each machine-learning model. Each boxplot represents the distribution of SHAP values across models for a given variable. The ten most influential predictors overall are displayed, ranked by their aggregated importance.

When analyzing model training using the combined dataset from all hospitals, it was observed that 66.5% of test set patients were not misclassified by any algorithm, while 11.5% were misclassified by all of them. A comparison of predictor value distributions between these two groups, using a paired *t*-test for normally distributed homoscedastic variables, revealed that age, mean corpuscular volume (MCV), and lymphocyte count rejected the null hypothesis at the 5% significance level. For variables with unequal variance between groups, comparisons were conducted using the non-parametric Mann-Whitney U test. At the 5% significance level, the null hypothesis was rejected for neutrophils, red blood cell count, respiratory rate, platelet count, and C-reactive protein (CRP). These findings are consistent with the variable importance profiles derived from Shapley values (Fig 3).

Together, these findings suggest that model errors are not randomly distributed across the population but are instead associated with specific clinical and laboratory profiles, motivating further stratification of the cohort.

### Cluster-specific insights

To further characterize population heterogeneity and assess its impact on predictive performance, we applied unsupervised clustering to identify clinically distinct patient subgroups.

**Fig 4:**
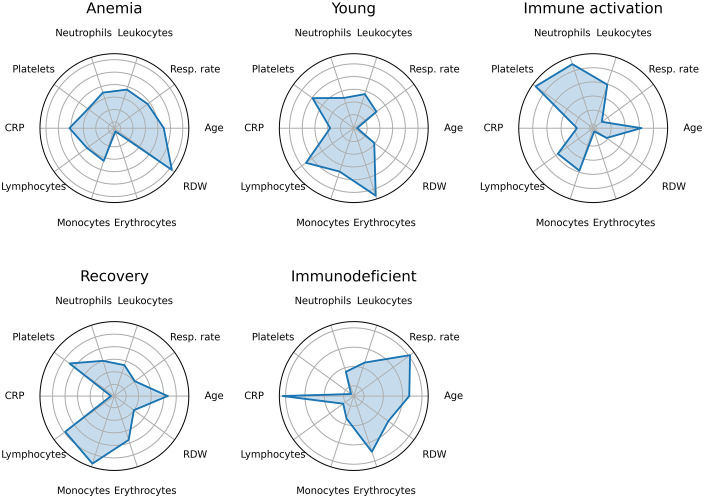
Cluster characterization radar charts. Radar charts depicting the mean standardized values of the ten most influential predictors (as identified by SHAP analysis) within each patient cluster. Standardization was performed using z-score normalization based on the overall cohort. Each chart summarizes the characteristic clinical profile of the corresponding cluster.

The training set was split into five clusters by k-means. This number was chosen by the elbow method, through minimization of the weighted cumulative squares sum (WCSS). Fig 4 illustrates this clusters’ profiles in radar charts created using the ten most important predictors according to the Shapley values analysis.

Analysis of the radar plots and group-wise mean values of the normalized variables indicates that the first cluster, denominated “Anemia”, comprising 529 patients, is characterized by individuals with higher average age (+0.23), normal respiratory rate (−0.09), C-reactive protein (+0.05), and leukocyte count (−0.12). This cluster also presents a slight reduction in neutrophils (−0.24), a moderate decrease in platelet count (−0.43), and a pronounced deficiency in red blood cells (−1.61), accompanied by a substantial increase in red cell distribution width (RDW) (+1.10). This profile is indicative of a group of anemic patients with marked anisocytosis (elevated RDW). The observed mortality rate within this cluster was 79.58%.

“Young” represents the group with the lowest mean age (−0.69), with slightly reduced respiratory rate (−0.21), and near-average values for leukocytes (−0.06), neutrophils (−0.13), C-reactive protein (−0.30), and RDW (−0.26). This group exhibits elevated lymphocyte (+0.39) and red blood cell (+0.63) counts. Comprising a total of 1,310 patients, this cluster appears to represent a relatively healthy population with a robust immune response and low levels of systemic inflammation. The observed mortality rate for this group was 23.20%.

Consisting of 1,286 patients, the third cluster is distinguished by an older age profile (+0.42), elevated leukocyte (+0.39), neutrophil (+0.69), and platelet counts (+0.75), as well as moderately increased lymphocytes (+0.36). C-reactive protein (−0.02) and red blood cell count (−0.20) are near the average. This profile is indicative of pronounced immune activation, which could reflect either a more widespread infectious process or a well-functioning immune response. The mortality rate in this group was 53.49%.

“Recovery” cluster is the smallest, including 295 patients with slightly increased age (+0.26), reduced respiratory rate (−0.18), and normal leukocyte (−0.06) and neutrophil (+0.00) counts. It is characterized by elevated lymphocytes (+0.38) and monocytes (+0.54), along with markedly reduced C-reactive protein (−0.53). This group exhibited the lowest mortality rate among all clusters, at 22.37%.

The fifth cluster comprises 964 patients with moderately elevated age (+0.17) and substantially increased respiratory rate (+0.53), alongside reduced leukocyte (−0.35), neutrophil (−0.60), lymphocyte (−0.92), and monocyte (−0.65) counts. These patients also show elevated C-reactive protein (+0.57) and slightly increased red blood cell count (+0.25). This profile suggests a systemic inflammatory state with respiratory compromise, potentially indicative of sepsis or respiratory failure. The associated mortality rate was 73.92%. These findings are summarized in Table 3.

**Table 3:** Qualitative description of clusters.

Id	Cluster Name	Qualitative Description	Mortality Rate
0	Anemia	Patients with severe anemia (markedly reduced red blood cell count) and high variability in red blood cell size (elevated RDW). Moderate age and mild inflammation (CRP).	High (79.58%)
1	Young	Young group with hematological and inflammatory parameters within normal ranges. Slightly elevated red blood cells and lymphocytes, suggesting overall good health.	Low (23.20%)
2	Immune Activation	Inflammatory profile characterized by elevated leukocytes, neutrophils, platelets, and lymphocytes. May reflect a robust immune response or systemic infection.	Moderate (53.49%)
3	Recovery	Profile with elevated lymphocytes and monocytes and significantly reduced CRP levels. May indicate recovery from a recent infection, with persistence of immune cells but absence of acute inflammation.	Low (22.37%)
4	Immunodeficient	Group showing significant immunosuppression (global reduction of white blood cells), elevated systemic inflammation (CRP), and increased respiratory rate. Indicative of clinical severity.	High (73.92%)

**Fig 5:**
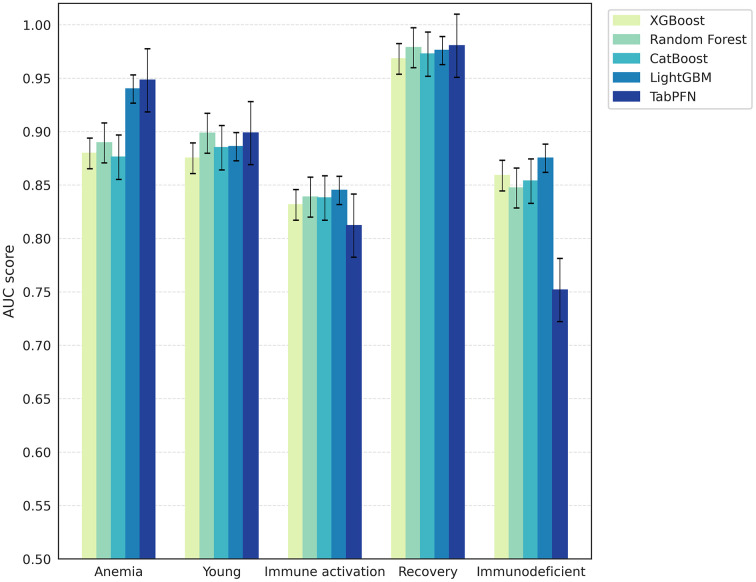
Within-cluster algorithmic performance comparison. Bar plots showing the predictive performance of each machine-learning algorithm within individual patient clusters, evaluated using 10-fold cross-validation. Bars indicate mean performance across bootstrap samples, and error bars represent 95% confidence intervals.

The five algorithms were retrained on data specific to each patient cluster using 10-fold cross-validation, and their predictive performance was assessed based on the mean area under the ROC curve (Fig 5). Analysis of variance (ANOVA) test revealed significant heterogeneity in performance across clusters (F = 73.182316, *p* < 0.001). Notably, the best performance occurred in the “Recovery” cluster, which had the smallest sample size (295) and low mortality rate (23.37%). Meanwhile, in the “Immune Activation” cluster (n = 1286, mortality rate = 53.49%), all algorithms underperformed.

Within a 95% confidence interval, all algorithms exhibited statistically equivalent performance in the “Young,” “Immune Activation,” and “Recovery” clusters—subgroups also characterized by the lowest mortality rates. In contrast, the “Anemia” cluster demonstrated significantly superior performance from TabPFN and LightGBM. Notably, in the “Immunodeficient” cluster, TabPFN exhibited a mean AUC of 0.75 (95% CI: 0.72–0.78), markedly lower than the other algorithms, which achieved mean AUCs near 0.85. These results underscore the variation in algorithmic performance across clinically distinct subpopulations, highlighting the necessity of incorporating population-specific characteristics into model selection for clinical prediction tasks and suggesting that future studies should explicitly evaluate subgroup-specific performance rather than relying solely on aggregate metrics.

## Discussion

This study demonstrates that all five evaluated algorithms achieved consistently high performance across diverse clinical settings, with TabPFN emerging as the top-performing and most stable model in most scenarios. Notably, TabPFN required no hyperparameter tuning and exhibited the lowest performance variance across hospitals. These results corroborate those of Hollmann et al. (2025), who found TabPFN to outperform several benchmark models, including Random Forest, XGBoost, Catboost, and LightGBM, on datasets with up to 10,000 samples [11].

Our findings on predictive performance are consistent with those reported by Savalli and Wichmann using the same database [12,13]. Additionally, the variable importance profiles align with those identified by Smith and Alvarez (2021), who highlighted age, lymphocyte count, and neutrophil count as key predictors of covid-19 mortality [14]. The recurrence of these variables across different analytical frameworks supports the robustness of our approach. Importantly, the observation of substantial predictive divergence among models with similar global performance metrics contributes new empirical evidence to the discourse on algorithmic multiplicity [6–8,10,15]. This reinforces the notion that even models with similar architectures can produce markedly different individual-level predictions, as evidenced by low R² values.

A major strength of this study lies in its multifaceted evaluation framework, which integrates algorithm performance across multiple hospitals, individual prediction consistency, algorithmic similarity analysis, variable importance via SHAP values, and subgroup-based performance comparison. A key novelty is the extension of algorithmic multiplicity analyses to a country as large and socio-demographically diverse as Brazil. This broader context enables models to capture complex, context-specific interactions and provides a more realistic assessment of model behavior under heterogeneous clinical conditions.

Nevertheless, several limitations must be considered. First, the analysis was restricted to a single national dataset collected during the early phase of the covid-19 pandemic, which may limit generalizability to subsequent viral variants or international populations. Second, although preprocessing and training procedures were standardized to ensure fair comparison, the interpretability of some models—most notably TabPFN—remains limited due to incompatibility with widely used explainability tools. Third, while the unsupervised clustering approach proved useful for delineating patient subgroups, it may be sensitive to initialization and hyperparameter choices, warranting caution when extrapolating clinical interpretations from these clusters.

An additional limitation is the restricted scope of evaluated models. The analysis was limited to five ML algorithms and therefore does not capture the full spectrum of available approaches. As a result, conclusions regarding algorithmic multiplicity may be primarily applicable to high-capacity, non-linear predictors. Future research should systematically expand the model space to examine how architectural assumptions, learning objectives, and inductive biases contribute to predictive multiplicity. Such comparative efforts would support the development of more general guidelines for model selection, ensemble construction, and clinical deployment in heterogeneous healthcare settings.

The error mechanisms identified in this work highlight several avenues for developing more equitable ML models. Future research should prioritize strategies to mitigate biases arising from predictive multiplicity, including dual-prioritization bias correction through subgroup-specific modeling [16] and ensemble methods guided by multi-objective optimization to jointly balance accuracy and fairness [17]. Meta-learning approaches, which leverage prior knowledge to accelerate adaptation to new tasks, also hold promise for deployment in dynamic and heterogeneous clinical environments [18]. Moreover, advances in interpretability—particularly for complex architectures such as TabPFN—and validation across multiple institutions and broader patient populations will be critical for improving model transparency and generalizability. Ultimately, adaptive ensemble systems that explicitly incorporate fairness and stability metrics may offer a principled pathway toward more equitable and personalized clinical decision-support tools.

## Conclusions

Using a large, multicentric cohort of hospitalized covid-19 patients in Brazil, this study provides empirical evidence that ML models with comparable overall performance can produce substantially divergent predictions at both the individual and subgroup levels when applied to mortality prediction.

Despite achieving similarly high aggregate AUC values, the evaluated algorithms exhibited heterogeneous probabilistic outputs, distinct classification patterns, and marked variability in performance across clinically meaningful patient subgroups. These findings indicate that reliance on global performance metrics alone may obscure clinically relevant differences in model behavior and may inadvertently amplify inequities when models are deployed in heterogeneous patient populations.

Although focused on covid-19 mortality, the methodological insights presented here are broadly applicable to other clinical prediction tasks and healthcare contexts. As ML continues to be integrated into clinical decision-making, systematically characterizing and addressing algorithmic multiplicity may support the development of more transparent, reliable, and equitable predictive systems.

## Supporting information

S1 Table Characteristics of participating hospitals included in the study. (DOCX)

S2 Table Model Hyperparameters. Hyperparameters for each model, selected using Bayesian optimization on the aggregated dataset.
(DOCX)

S3 Table Performance metrics by model. Detailed performance metrics for each model evaluated on the aggregated dataset.
(DOCX)
