# Supplementary material for: Predictive divergence in machine learning models for clinical mortality risk: A multicohort study of covid-19 patients
Source: PLoS One. 2026 Mar 6;21(3):e0344354. doi: 10.1371/journal.pone.0344354 (PMC12965533; doi:10.1371/journal.pone.0344354)
Supplement: S1 Table — (DOCX) [file pone.0344354.s001.docx]

| **Hospital** | **CNES** | **State** | **Type of legal entity** | **Level of government** | **Type of hospital** | **Delivery** | **Teaching and research** | **N. beds** |
| --- | --- | --- | --- | --- | --- | --- | --- | --- |
| Hospital Santa Julia | 2018055 | AM | Business entity | State | General hospital | Tertiary care | Has no teaching activity | 176 |
| Hospital Português da Bahia | 0004251 | BA | Not-for-profit | State and municipal | General hospital | Tertiary care | Teaching Hospital | 344 |
| Hospital Estadual de Luziânia | 2340429 | GO | Government-owned corporation | State | General hospital | Tertiary care | Has no teaching activity | 60 |
| Hospital Santa Casa de São Paulo | 2688689 | SP | Not-for-profit | State | General hospital | Tertiary care | Teaching Hospital | 568 |
| Hospital Moinhos de Vento | 3006522 | RS | Not-for-profit | Municipal | General hospital | Tertiary care | Has no teaching activity | 445 |
