# Supplementary material for: Predictive divergence in machine learning models for clinical mortality risk: A multicohort study of covid-19 patients
Source: PLoS One. 2026 Mar 6;21(3):e0344354. doi: 10.1371/journal.pone.0344354 (PMC12965533; doi:10.1371/journal.pone.0344354)
Supplement: S2 Table — Hyperparameters for each model, selected using Bayesian optimization on the aggregated dataset. (DOCX) [file pone.0344354.s002.docx]

| **Algorithm** | **Hyperparameters** |
| --- | --- |
| **CatBoost** | depth: 10, iterations: 536, l2_leaf_reg: 3.871280044, learning_rate: 0.4473725997 |
| **LightGBM** | colsample_bytree: 0.512337107, learning_rate: 0.02722892459, max_depth: 21, min_child_samples: 47, n_estimators: 312, num_leaves: 100 |
| **RandomForest** | criterion: 'entropy', max_depth: 1030, max_features: 'sqrt', min_samples_leaf: 0.0005036636679, min_samples_split: 0.00421692932, n_estimators: 2 |
| **XGBoost** | colsample_bytree: 0.6890442998, gamma: 0.0002189911326, learning_rate: 0.09448045665, max_depth: 13, min_child_weight: 1, n_estimators: 700, reg_alpha: 0.7, reg_lambda: 0.1 |
