# Supplementary material for: Predictive divergence in machine learning models for clinical mortality risk: A multicohort study of covid-19 patients
Source: PLoS One. 2026 Mar 6;21(3):e0344354. doi: 10.1371/journal.pone.0344354 (PMC12965533; doi:10.1371/journal.pone.0344354)
Supplement: S3 Table — Detailed performance metrics for each model evaluated on the aggregated dataset. (DOCX) [file pone.0344354.s003.docx]

| **Model** | **AUC** | **Accuracy** | **Precision** | **Recall** | **F1 Score** |
| --- | --- | --- | --- | --- | --- |
| **CatBoost** | 0.849 | 0.793 | 0.636 | 0.636 | 0.636 |
| **LightGBM** | 0.863 | 0.794 | 0.627 | 0.682 | 0.653 |
| **Random Forest** | 0.861 | 0.789 | 0.611 | 0.711 | 0.658 |
| **TabPFN** | 0.863 | 0.796 | 0.663 | 0.578 | 0.617 |
| **XGBoost** | 0.854 | 0.792 | 0.635 | 0.636 | 0.636 |
